# Medicinal plants diversity among the oromo community in heban-arsi district of Ethiopia used to manage human and livestock ailments

**DOI:** 10.3389/fphar.2024.1455126

**Published:** 2024-09-19

**Authors:** Geritu Nuro, Ketema Tolossa, Mahlet Arage, Mirutse Giday

**Affiliations:** Aklilu Lemma Institute of Pathobiology, Addis Ababa University, Addis Ababa, Ethiopia

**Keywords:** medicinal plants, traditional knowledge, traditional healers, general informants, Heban-Arsi district, Ethiopia

## Abstract

**Introduction:**

Medicinal plants are commonly employed mainly due their accessibility, affordability and potency. However, medicinal plants and the associated knowledge are disappearing at an alarming rate due to natural and anthropogenic causes and thus a need for their proper documentation conservation. This study was performed to document traditional knowledge related to use of medicinal plants in Heban-Arsi district, West-Arsi Zone, Oromia Regional State, Ethiopia.

**Methods:**

Interviews were conducted with 185 informants to identify medicinal plants used in traditional therapies in the study area. Informant consensus factor (ICF), rank order priority (ROP) values were computed, and preference ranking exercises were performed to assess the relative importance of medicinal plants. Descriptive and inferential statistics were used to measure and compare medicinal plants knowledge between social groups.

**Results:**

A total of 120 medicinal plants were identified for being used to treat different human and animal illnesses in the study area. Most of the medicinal plants (76.4%) were uncultivated ones obtained from different habitats. Leaf was the most frequently used plant part constituting 62.6% of preparations. Oral was the most commonly used route of remedy administration (46%) in treating diseases. Gastrointestinal ailments category had the highest ICF value (0.83). In the study area, the highest rank order priority (ROP) values were recorded for *Dombeya torrida* (J.F. Gmel.), *Artemisia absinthium* L., *Balanites aegyptiaca* (L.) Del., *Combretum pisoniiflorum* Klotzsch) Engl., *Celtis africana* Burm. f, *Ocimum gratissimum* L. and *Lagenaria* sp. for their uses against snake poison, tuberculosis, liver disorder, stomachache, tuberculosis, febrile illness and liver disorder, respectively, each scoring a value of 100. Significant differences in medicinal plant use knowledge were recorded between male and female informants of different educational level, age and experience. Anthropogenic factors were the primary threats to medicinal plants in the area.

**Conclusion:**

The study area was found to be rich in medicinal plants that are useful in treating a wide range of human and animal illnesses. In future pharmacological and phytochemical investigations, priority needs to be given to medicinal plants of the highest ROP values and those that were reported against ailment categories scoring the highest ICF values.

## 1 Introduction

Historically, humans have relied on nature to provide for their basic needs, including the use of plants as medicine to treat a variety of illnesses (Cragg and Newman, 2013). The World Health Organization estimates that 80% of people in developing countries receive their medical care from traditional medicines, most of which are made from plants (Rasul, 2018) due to the fact that the majority of individuals residing in developing nations inhabit rural regions with limited availability of modern healthcare facilities (World Health Organization, 2001). Furthermore, due to problems related to antimicrobial resistance to chemical drugs, affordability and accessibility, traditional medicines are still considered important and sustainable sources of treatment (Patwardhan, 2005). Researchers are thus compelled to explore compounds with potential antimicrobial properties derived from natural sources, specifically from traditionally utilized medicinal plants (Dhama et al., 2014; Dilbato et al., 2019). Medicinal plants are rich sources of pharmaceutical and therapeutic products because they can synthesize chemicals through secondary metabolism that have antibacterial properties (Reddy et al., 2022). Plant products consist of a variety of constituents that are chemically complex mixtures with various potential targets and modes of action (Mickymaray, 2019; Efferth and Koch, 2011). The distinct attributes possessed by plants, such as their abundant diversity of chemical compounds (Gurib-Fakim, 2006), established therapeutic effectiveness of their extracts in longstanding traditional medical practices worldwide (Yang et al., 2014), their widespread availability, and the potential for mutually synergetic effects between phytochemicals (Caesar and Cech, 2019), have positioned them as the preferred choice especially for addressing the issue of drug resistance.

Traditional medicine has been practiced in Ethiopia since antiquity and hence it has permeated all social groups, and continues to be important to both human and animal health (Giday et al., 2003; Kefalew et al., 2015). This could be because it is associated with spiritual, historical, or social values, or because the community believes that medicinal plants can heal (Bishaw, 1991). It is postulated that approximately 80% of the rural populace in Ethiopia and roughly 90% of their livestock depend on traditional medicine for the fulfillment of fundamental healthcare requirements (Abebe, 2001). Due to the heterogeneous nature of its ethnic communities, the extensive utilization of conventional therapeutic plant medicine throughout an extended duration, and diverse topography, Ethiopia harbors an extensive repository of traditional medicinal plants and indigenous wisdom. So far, 887 plant taxa have been reported to be used to treat various health problems in the country (Mesfin et al., 2009; Kidane et al., 2014).

Like other developing countries, Ethiopian plant resources, including medicinal plants (Asfaw and Tadesse, 2001) and related indigenous knowledge have been disappearing at an alarming rate (Regassa, 2013; Mesfin et al., 2014) mainly because of habitat loss, forest deforestation, excessive use of forest resources, population growth, poverty and illegal trade (Giday et al., 2003; Lulekal et al., 2008; Megersa et al., 2013; Tolossa et al., 2013). The predominantly oral culture of traditional knowledge transfer is also considered to have contributed to the deterioration of traditional medical knowledge in Ethiopia. (Getahun, 1976). Traditional healers in the country often share their knowledge only with their eldest sons and most of their traditional information is kept secret (Jansen, 1991). Over the past decade, ethnobotanical research in Ethiopia has been increasing, and several studies have documented the use of plants as medicines for various human and animal diseases. (Chekole, 2017; Kebebew and Mohamed, 2017; Agize et al.*,* 2022; Tahir et al., 2023). In the Heban-Arsi district, the local communities are known for using medicinal plants to treat various human and animal diseases. However, there has been no comprehensive ethnobotanical study conducted so far that produced an exhaustive list of medicinal plants used in the district. A better medicinal plants diversity is expected in the district as there is a protected natural forest in the area known by the name Munesa Forest and wide practice of using medicinal plants to manage different human and livestock ailments. Thus, the aim of this study was to investigate and record the traditional use of medicinal plants by the residents of Heban-Arsi district and generate baseline data for ongoing studies aimed at developing plant-based products in the country for the treatment of human and animal ailments.

## 2 Materials and methods

### 2.1 Study area

**FIGURE 1:**
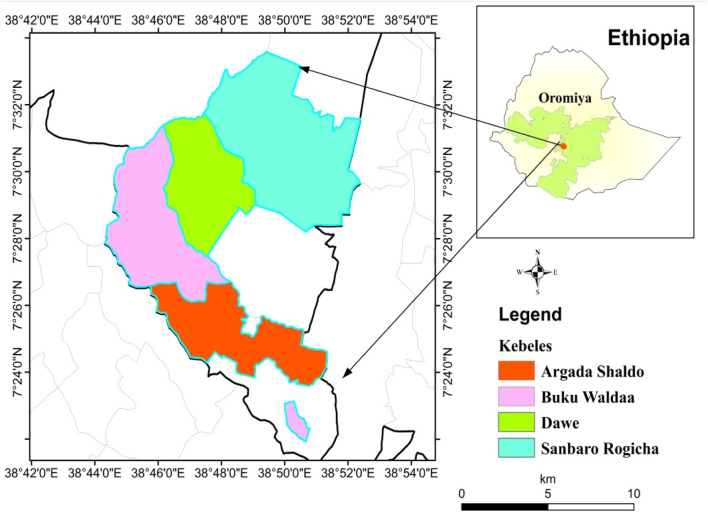
Map of Heban-Arsi district showing sampled kebeles.

The study was conducted in Heban-Arsi district, West-Arsi Zone of Oromia Regional State, southeastern Ethiopia (Figure 1). The district is located at 7° 9′–7° 42′N and 38° 25′–38° 54′E, with an elevation ranging from 1,500 to 3,000 m above sea level (m.a.s.l.) (Heban-Arsi district Agricultural Development Office, unpublished data). The annual average rainfall of the study area is 825 mm and the mean annual temperature is 19°C (Heban-Arsi district Agricultural development Office, unpublished data). The district has three major climatic zone: highland (2,000–3,000 m.a.s.l.) with annual temperature of 10°C–18°C), midland (1,800–2,000 m.a.s.l.) with annual temperature of 15°C–22°C), and lowland (1,500–1,600 m.a.s.l.) with annual temperature of 19°C–27°C (Heban-Arsi district Agricultural development Office, unpublished data). The district rainy period is from March to mid-September with peak in August. The district has nine kebeles (the smallest government administrative structure in Ethiopia) and three towns. According to the Central Statistics Agency (CSA) of Ethiopia, there are 75,831 people living in the district, of which 41,103 are men and 34,728 are women (CSA, 2007). Out of the total population, 94% are rural dwellers while 6% live in towns. In the district there is protected natural forest area known as Munesa Forest Enterprise. This forest is dwindling in terms of both vegetation coverage and species diversity because of land clearing for agricultural use, local timber production, collection of firewood and the production of charcoal by the local community. The district has nine health posts and three health centers. In terms of veterinary facilities, there are four veterinary clinics in the district. The district has a large population of livestock, including goats (60, 980), cattle (43,812), sheep (19,102), donkeys (19,468), horses (982), mules (919) and poultry (62,143) (Heban-Arsi district Agricultural development Office, unpublished data). The top-ten human diseases in the district are gastrointestinal disorders, skin infections, intestinal parasites, malaria, liver disorders, pneumonia, snakebite poisoning, tonsillitis, hemorrhoids and diabetes (Heban-Arsi district Healthcare Office unpublished data). Diseases that affect animals include bloat, mastitis, bovine TB, anthrax, pasterollisis, and dermatophilosis, external and internal parasites (Heban-Arsi district Agricultural development Office, unpublished data).

### 2.2 Study design, study sites and informants selection

A reconnaissance survey was conducted in Heban-Arsi district in September 2022 with the purpose of selecting study kebeles and informants. Accordingly, four kebeles distributed across two agro-ecological zones of the district, and known for rich plant-based traditional medical practice were purposively identified for the study with the help of elders and local authorities. Argada Shaldo and Sanbaro Rogicha kebeles were from midland agro-climatic zone and Buku Waldaa and Dawe kebeles were from lowland agro climatic zone. From each kebeles, two villages were selected for sampling (Table 1). For this study, a total of 185 informants were sampled using both random and purposive sampling methods. Of the total selected informants, 165 were general informants randomly sampled following procedures outlined in Kefalew et al. (2015) with minor modifications. The remaining twenty informants were traditional healers deliberately chosen based on suggestions provided by elders, local government officials, and health extension workers at every research site (Table 1).

**TABLE 1:** Study kebeles and sampled villages in the study district.

S.N.	Study kebele	Altitude (L)	Sampled villages	No. of sampled informants		
				GIs	THs	Total
1	Argada Shaldo	1701–2000 m.a.s	Koromi	21	3	24
			Argada	21	2	23
2	Sanbaro Rogicha	1600–1981 m.a.s	Rogicha	21	2	23
			Degaga	21	3	24
3	Buku Waldaa	1500–1550 m.a.s	Alembadaa	21	3	24
			Kimphe	21	2	23
4	Dawe	1501–1600 m.a.s	Langano	21	2	23
			Bishangaari	18	3	21
Grand total						185

Note: GIs, General informants; THs, Traditional Healers, m.a.s.l. = metre above sea level.

### 2.3 Ethnobotanical data collection

Ethnobotanical data was gathered between November 2022 and September 2023. Prior to the initiation of interviews, every informant who took part in this investigation was provided with an explanation regarding the objectives of the study and assured of the utmost responsible and judicious handling of the information. The main methodologies employed for the collection of ethnobotanical data encompassed semi-structured interviews and field visits (Alexiades, 1996; Cotton, 1996; Martin, 1995). The data collection process was facilitated through the utilization of a checklist containing predetermined interview questions. The interviews were conducted exclusively in the Afan Oromo language, the most widely spoken language in the area. During interviews, information on demographic characteristics of respondents, local names of medicinal plants used, specific parts of plants utilized, ailments against which reported medicinal plants were employed, methods of remedies preparations and administrations, prescribed doses, habits and habitats of the medicinal plants, utilization of other ingredients or additives, any notable adverse effects of remedies, use of antidotes, taboos and beliefs associated with the collection and use of plants, sources of knowledge, and methods of indigenous knowledge transferred was gathered. Information concerning current risks to medicinal plants and associated knowledge as well as any traditional preservation techniques was also obtained following known methodologies (Alexiades, 1996; Balick and Cox, 1996; Cotton, 1996). Field walks with informants were also made to note the morphology and habitats of the claimed medicinal plants (Alexiades, 1996; Cotton, 1996) in the study area. Plant specimens were collected, pressed, dried, and brought to Addis Ababa University, Aklilu Lemma Institute of Pathobiology (ALIPB). Voucher specimens were identified by botanists at ALIPB and the National Herbarium, Addis Ababa University (AAU), checked for their latest accepted names, on the website Plants of the World Online, and deposited for future reference.

### 2.4 Data analysis

Ethnobotanical data were compiled in Microsoft Excel spreadsheets and analyzed using SPSS version 20. The most useful information collected about medicinal plants was summarized using descriptive statistical methods, such as frequency and percentage. Preference ranking exercises were conducted on selected medicinal plants based on Martin (1995) to identify the most preferred ones. Selected key informants assigned values from 0 to 5 with 5 given for the most preferred and 0 for the least preferred plant species against certain diseases. The values were then summed, and ranks were assigned to each plant species. Informant consensus factor (ICF) was computed to determine the most culturally important human and livestock ailment categories in the study area and identify potentially effective medicinal plant species. Reported diseases were grouped into categories based on the International Classification of Diseases (ICD-10) by the World Health Organization (2011). Informant consensus factor was calculated using the formula, ICF = (n_ur_ − n_t_)/(n_ur_ − 1), where n_ur_ is the number of use reports for a particular use category and n_t_ is the number of taxa used for a particular use category by all informants (Heinrich et al., 1998). Informant Consensus index values range between 0 and 1. A high value (close to 1) indicates that relatively few species are used by a large proportion of healers. High ICF value is indicative of the plant’s cultural importance and may show its high relevance for pharmacological studies. The relative healing potential of each reported medicinal plant used against human and livestock ailments were evaluated using an index known as rank order priority (ROP) computed based on fidelity level (FL) values, which were calculated for all reported medicinal plants using the formula: FL = Ip/Iu × 100 (Friedman et al., 1986), where Ip is the number of informants who reported the utilization of medicinal plants against a specific ailment and Iu is the total number of informants who mentioned the same plant against any ailment. Plants exhibiting comparable FL values known by varying numbers of informants could exhibit differences in their medicinal efficacy. Therefore, correlation index called relative popularity level (RPL) was established to calculate rank order priority (ROP) value obtained by multiplying FL value by RPL to distinguish medicinal efficacy of plants with similar FL values. The plants were divided into “popular” (with RPL value of 1) and “unpopular” (with RPL value of less than1). “Popular” plants are cited by half or more of the highest number of informants who reported a given plant against any ailment, whereas “unpopular” plants are cited half by less than half of the maximum number of informants who cited a given plant against any ailment. Actual RP value for “unpopular” medicinal plant is calculated by dividing its number of informant citations by the maximum number of citation obtained for a given plant in the list.

### 2.5 Ethical consideration

The study proposal was reviewed and approved by the Addis Ababa University, Aklilu Lemma Institute of Pathobiology Institutional Research Ethics Review Committee (ALIPB-IRERC). Permissions were obtained from West-Arsi zone, and study district and Kebeles administrative offices to carry out the fieldwork. Verbal consent was obtained before interviewing each informant and traditional healer.

## 3 Results

### 3.1 Socio-demographic profile of informants

Out of the total 185 informants involved in this study, 78.4% (n = 145) were males and 21.6% (n = 40) were females. The age of the informants in the study ranged from 20 to 89. Most of the informants were aged 41–60 years (45.4%; 84), followed by those aged 61–89 years (35.1%; 65) and aged 20–30 years (19.5%; 36). Regarding educational status of the informants, the majority (60.5%; 112) were illiterate and the remaining (39.5%; 73) were literate.

### 3.2 Diversity of medicinal plants used

**FIGURE 2:**
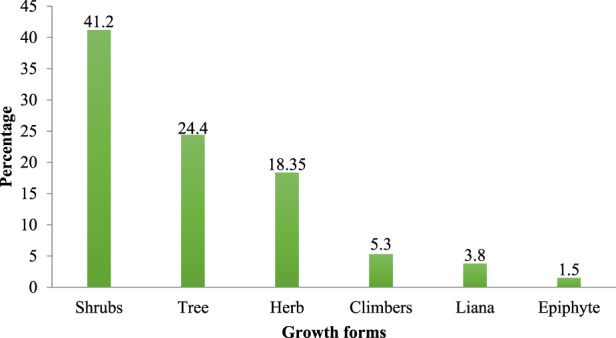
Growth forms of traditional medicinal plants.

The study found that 120 traditional medicinal plants taxa, distributed across 98 genera and 55 families, were used by the community in Heban-Arsi district (Supplementary Table 1). Of the total medicinal plants, 43.4% belong to the families Fabaceae (7.6%), Asteraceae (5.3%), Solanaceae (5.3%), Euphorbiaceae (3.8%), Lamiaceae (3.8%), Olacaceae (3.05%), Tiliaceae (3.05%), Moraceae (2.3%), Vitaceae (2.3%), Cucurbitaceae (2.3%), Rosaceae (2.3%) and Myrtaceae (2.3%), and the rest families cover 56.6% of the taxa. The reported medicinal plants had different growth forms including shrubs (41.2%), tree (24.4%), herbs (18.35%), climbers (5.3%), lianas (3.8%) and epiphytes (1.5%) (Figure 2).

### 3.3 Habitat of medicinal plants

The majority of medicinal plants (76.4%) used in the study district were reported to be uncultivated that were harvested from forests, rivers banks, grasslands, farm fields, farm edges, hills sides, roadsides, life fences and school compounds. Some were cultivated in home gardens (15%), and few others were harvested from both the wild and homestead gardens (8.6%).

### 3.4 Part of medicinal plants used in remedy preparation

**FIGURE 3:**
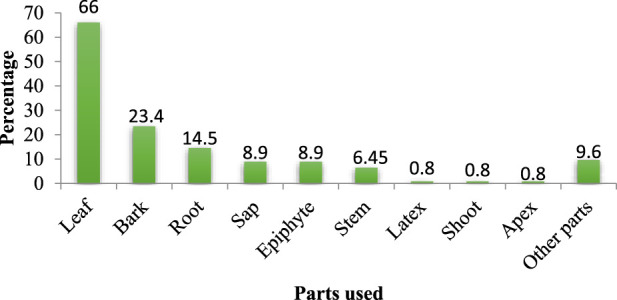
Proportions of medicinal plant parts used in remedy preparation.

Leaf was the most commonly used plant part accounting for 62.6% of the remedy preparations, followed by bark (22.14%) (Figure 3).

### 3.5 Plant remedies preparation methods

**FIGURE 4:**
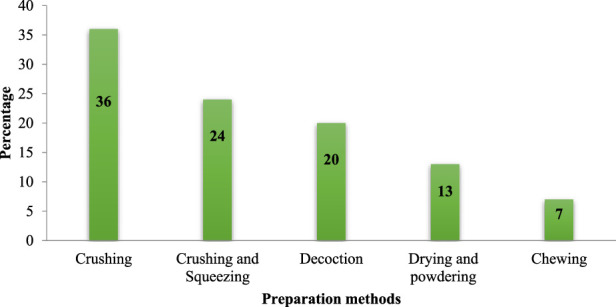
Proportions of plant remedies preparation methods.

Crushing and homogenizing in cold and clean water was the primary way of plant remedy preparation (36%), followed by crushing and squeezing (24%), decoction (20%), drying and powdering (13%) and chewing (7%), (Figure 4). Most remedies were prepared from freshly collected plant parts (89%) while some (11%) were prepared from dried plant parts.

### 3.6 Routes of remedy administration

**FIGURE 5:**
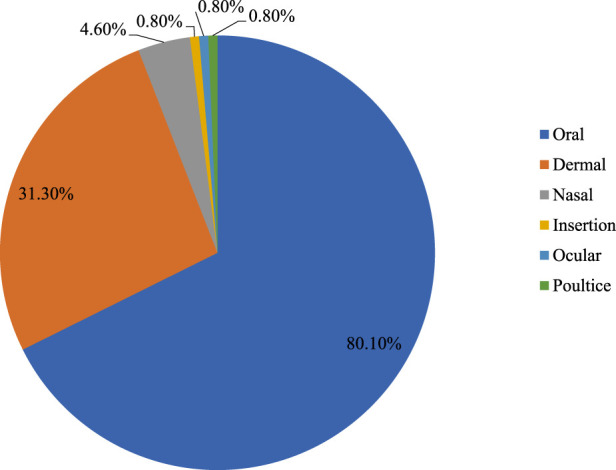
Routes of remedy administration.

Result revealed that oral application was the most common route of remedy administration (80.1%) followed by external/dermal (32.1%) and nasal (4.6%) application. Few remedies were reported to be administered by insertion into deep wound (0.8%) and ocular (0.8%) (Figure 5).

### 3.7 Dosage forms, side effects and use of antidotes

The majority of traditional healers (81%) in the study area understood the effects of overdose use of the medicinal plants on the health of patients. They determined the dosages based on patient’s sex, age, presence and absence of pregnancy, body condition, nature of the ailment treated and the route of administration. Water glass, teacup, coffee cup, teaspoon, bottle cap, handful, and between two tips of fingers were used to determine dosages. Traditional healers advised their patients to drink fresh milk, coffee, yoghurt, roasted barley juice, butter and honey as antidotes during adverse effects. For example, leaves of *Discopodium penninervum* Hochest. were chewed and swallowed after orally taking *Gymnathemum auriculiferum* (Hiern) Isawumi decoction for the treatment of sexually transmitted diseases.

### 3.8 Frequently reported human and livestock ailments in the study district

In the district, 54 human and 25 livestock ailments were recorded during interviews. The highest cited human ailments in the district were gastrointestinal complaints (157; 84.9%), skin disorders (98; 52.9%), infectious and parasitic diseases (liver disorders and internal parasite) (82; 44.3%), and malaria (71; 38.3%), while gastrointestinal disorders (bloat, stomachache and diarrhea) (130; 70.2%) and bacterial infections (anthrax and pasterollosis) (27; 14.6%) were the highest cited diseases of livestock.

### 3.9 Preference ranking of selected medicinal plants

#### 3.9.1 Preference ranking of medicinal plants used for treatment of human diarrhea

Result of preference ranking exercise conducted on medicinal plants that were used against diarrhea, the most commonly cited ailment in the study area, showed that Syzygium guineense (willd.) DC, Ximenia americana L., and Gymnathemum amygdalinum (Del.) Sch.Bip. ex Walp. were the most preferred plants (Table 2).

**TABLE 2:** Results of preference ranking of ten medicinal plants reported for treating human diarrhea in the study area.

Medicinal plants used for treatments of diarrhea	Informants designated A to J										Total	Rank
	A	B	C	D	E	F	G	H	I	J		
*Withania somnifera (*L.) Dunal	3	3	3	4	4	5	4	2	5	3	29	7
*Allophylus abyssinicus* Hochst. ex Benth	3	2	2	3	4	3	3	4	3	1	35	6
Vepris nobilis (Delile) Mziray	1	2	2	3	2	2	2	1	3	3	21	10
*Ziziphus spina-christi* (L.) Desf	2	1	2	2	3	3	2	2	3	3	23	9
*Ximenia americana* L	5	5	3	4	3	4	5	4	5	3	41	2
*Bersama abyssinica* Fresen	4	5	4	3	4	4	4	4	5	4	39	4
*Syzygium guineense* (willd.) DC.	4	5	3	5	5	3	4	5	5	4	43	1
Gymnanthemum amygdalinum (Del.) Sch.Bip. ex Walp	4	3	4	5	5	4	4	2	3	5	40	3
Combretum pisoniiflorum (Klotzsch) Engl	3	2	2	3	3	4	3	3	3	3	27	8
*Prunus africana (*Hook.f) Kalkman	4	3	4	5	5	4	4	3	2	4	38	5

#### 3.9.2 Preference ranking of medicinal plants used for treating livestock skin diseases

Preference ranking exercise conducted on nine medicinal plants that were reported to be used against skin diseases of the livestock in the study area was also conducted. The result showed that *Dodonaea viscosa* subsp. *angustifolia* (L.f.) J.G.West, *Calpurnia aurea* (Aiton) Benth and *Prunus africana* (Hook.f) Kalkman were the most preferred medicinal plants for the treatment of livestock skin diseases treatment in the area (Table 3).

**TABLE 3:** Results of preference ranking of ten medicinal plants reported for treating skin disease of livestock in the study area.

Medicinal plants used for the treatment of livestock skin diseases	Informants designated A to J										Total	Rank
	A	B	C	D	E	F	G	H	I	J		
Acokanthera schimperi (A.DC) Schweinf	1	2	3	2	2	3	2	4	2	4	25	9
Buddleja polystachya Fresen	3	4	3	4	5	2	3	3	3	2	32	6
Brucea antidysenterica JF. Mill	5	3	4	3	3	2	4	4	3	3	34	5
Dodonaea viscosa subsp. angustifolia (L.f.) J.G.West	5	4	4	5	4	5	5	5	5	4	46	1
Prunus africana (Hook.f) Kalkman	5	3	4	4	5	5	5	4	3	5	43	3
Maesa lanceolata Forssk	3	3	4	5	4	5	5	3	4	4	40	4
Calpurnia aurea (Aiton) Benth	4	4	4	5	5	4	5	5	3	5	44	2
Croton macrostachyus Hochst											30	7
Rhoicissus tridentata (L.f) Wild & Drummond	1	3	2	4	3	2	3	3	4	2	27	8

### 3.10 Informants consensus factor

Of the disease categories affecting human, gastrointestinal disorder category scored the highest ICF value (0.83), followed by infectious and parasitic diseases with ICF value of 0.80 and diseases of the skin and subcutaneous tissue diseases category with ICF value of 0.79 (Table 4).

**TABLE 4:** ICF values of different human ailment categories in Heban-Arsi district.

Disease category	n_t_	n_ur_	ICF
Injury, poisoning (snake bite, insect bite/spider, animal bite/rabies) and certain other consequences of external cause	12	44	0.74
Diseases of the skin and subcutaneous tissue (hair loss, dandruff, eczema, boils, itching, ringworm, wound)	21	98	0.79
Diseases of the reproductive and genitourinary system (urination difficulty, delayed menstruation, erectile problem, syphilis and gonorrhea)	8	33	0.78
Gastrointestinal disorders (constipation, diarrhea, stomachache, toothache, indigestion, abdominal pain)	27	157	0.83
Musculoskeletal diseases and nervous disorders (arthritis, rheumatism, epilepsy)	8	22	0.66
Psychospritual diseases	6	21	0.75
Respiratory ailments (TB, pneumonia, asthma) and tonsillitis	14	43	0.69
Sensory organs diseases (eye/ear diseases)	3	6	0.60
Infectious and parasitic diseases (jaundice, hepatitis, ascariasis, tapeworm)	17	82	0.80
Malaria, discomfort, fever/body pain/headache and sudden sickness	16	71	0.78
Diseases of the circulatory system (Hemorrhoids and body swellings, Anemia, blood pressure)	7	18	0.64
Neoplasms (tumor, breast cancer, skin cancer)	6	66	0.76
Endocrine and metabolic diseases (diabetes,)	3	9	0.66

Key: n_ur_: number of use citations in each disease category, n_t_: number of species used in each category.

The twenty-five livestock diseases reported in the district were categorized into nine disease categories for the calculation of ICF values. Accordingly, skin disorders category was the commonly reported livestock health problem with ICF of 0.89 (14 species, 130 use reports) followed by bloat and diarrhea, which is under the gastrointestinal disease category, was the second disease category with ICF of 0.88 (10 species, 80 use reports (Table 5).

**TABLE 5:** ICF values different livestock ailment categories in Heban-Arsi District.

Disease category	n_t_	n_ur_	ICF
Bacterial infection (anthrax, pasterollosis, black leg and mastitis)	6	27	0.80
Viral diseases (rabies, African horse sickness)	4	21	0.85
Pain (eye pain)	2	9	0.87
Gastrointestinal disorders (bloat, diarrhea, stomachache)	10	80	0.88
Respiratory disease (pneumonia, Bovine TB)	3	9	0.75
Reproductive and urinary tract infection (retained placenta, urinating difficulty)	3	17	0.87
Parasitic and protozoa causes (ticks, lice, leech and babesiosis, epizootic lymphangitis)	5	18	0.76
Skin diseases (dermatological, external injuries, wound, abscess and lumpy skin disease, swelling and tumor, & fungal diseases)	14	130	0.89
Evil eye	3	11	0.80

Key: n_ur_: number of use citations in each disease category, n_t_: number of species used in each category.

### 3.11 Rank order priority values

Results show that the plants *Dombeya torrida* (J.F. Geal.) Bamps, Artemisia absinthium *L.*, *Balanites aegyptiaca* (L.) Del., Combretum pisoniiflorum (Klotzsch) Engl., *Celtis africana* Burm. f, *Ocimum gratissimum* L. and *Lagenaria* sp. scored the highest ROP values (100 for each plant) for their uses to treat snake poison, tuberculosis, liver disorder, stomachache, tuberculosis, febrile illness and liver disorder, respectively (Table 6).

**TABLE 6:** Medicinal plants in the study area scoring an RPO value of above 90.

No.	Medicinal plants	Disease type	NP	N	FL	RPL	ROP
1	*Dombeya torrida* (J.F. Gmel.) Bamps	Snake poison	57	57	100	1	100
2	*Artemisia absinthium* L	Tuberculosis	47	47	100	1	100
3	*Balanites aegyptiaca* (L.) DelDC.	Liver disorder	46	46	100	1	100
4	Combretum pisoniiflorum (Klotzsch) Engl	Stomachache	40	40	100	1	100
5	*Celtis africana* Burm. f	Tuberculosis	38	38	100	1	100
6	*Ocimum gratissimum* L	Febrile illness	36	36	100	1	100
7	*Lagenaria* sp	Liver disorder	32	32	100	1	100
8	*Acokanthera schimperi* (A.DC). Schweinf	Malaria	61	63	96.8	1	96.8
9	*Phytolacca dodecandra* L ‘Herit	Rabies	49	51	96	1	96
10	*Syzygium guineense* (willd.)DC	Stomach ache	52	56	92.8	1	92.8

Note: FL, fidelity level; RPL , relative popularity level; ROP , rank order priority; Np = number of informants who independently cited the importance of a species for treating a particular disease; N = total number of informants who reported the plant for any given disease.

### 3.12 Ways of traditional medical knowledge acquisition

Acquisition of traditional medicinal knowledge happens in multiple ways. Most of (167, 90.3%) informants said they acquired traditional healing knowledge through their family routes orally. The remaining (18, 9.7%) informants confirmed acquiring their healing knowledge from different sources through observation, friends and following the activities of traditional healers.

### 3.13 Comparison of medicinal plants knowledge between social groups

A significant difference (*p* < 0.05) was observed between the mean numbers of medicinal plants claimed by traditional healers and general respondents with traditional healers reporting higher number of medicinal plants than the general respondents. Further comparison of knowledge showed that there was significant difference between the mean numbers of medicinal plants reported by males and females (*p* < 0.05) with males reporting a higher than females. There was also a significant difference (*p* < 0.05) between the mean numbers of medicinal plants reported two age groups and two informants groups of different educational level. Older (above 40 years old) and illiterate informants reported more mean numbers of medicinal plants than their respective younger and literate informants (Table 7).

**TABLE 7:** Comparison of medicinal plants knowledge between different social groups in the study area (n = 185).

Social group		Number of informants	Mean no of medicinal plants reported	*p*-value
Profession	Traditional healers	20	7.1	0.0001^a^
	General informants	165	3.2	
Gender	Male	145	5.9	0.0035^a^
	Female	40	3.1	
Ages	20–40	36	2.1	0.0001^a^
	41–89	149	5	
Educational level	Illiterate	112	3.12	0.007^a^
	Literate	73	1.5	

^a^ shows significant difference between groups with *p*-value <0.05.

### 3.14 Threats against medicinal plants and traditional medical knowledge in the study area

The present study revealed a number of threats against medicinal plants in the study district. The most frequently cited threat was the expansion of agriculture into forest area and grasslands, which was reported by 55% of the respondents, followed by production of timber, unsustainable fuelwood collection and charcoal making, which accounted for 35% of the informants. Frequent drought was also reported by 10% of the informants as factor responsible for degradation and extinction of medicinal plants. Factors responsible for the depletion of traditional medical knowledge related to use of medicinal plants in the area include lack of interest by the younger generation to learn and practice it (14%), cultural changes and modernization (20%), the oral culture of medicinal plant knowledge transmission (14%), the secrecy surrounding the traditional medicine (25%), and the migration of youth from rural areas to cities in search of employment opportunities (27%).

## 4 Discussion

The fact that high diversity of medicinal plant are used in the study area indicates the high dependence of people on medicinal plants in the day-to-day management of a wide range of human and livestock diseases and this might be related to easy accessibility, cultural acceptability and affordability of medicinal plants, and the associated services. In the study area, Fabaceae contributed higher number of medicinal plants as compared to other families. Other studies conducted elsewhere in Ethiopia also reported the high contribution of Fabaceae to local medicinal flora (Fenetahun et al., 2017; Kidane et al., 2018; Gijan and Dalle, 2019). Shrubs contributed the majority of medicinal plants used in the study area, which could be related to their year round availability and ease of harvesting. The dominance of shrubs was also reported in other ethnobotanical studies conducted elsewhere in the country (Lulekal et al., 2008; Tolossa et al., 2013; Gijan and Dalle, 2019). All medicinal plants documented from the current study area were found to have same or similar medical application in elsewhere in Ethiopia and other parts of the world (Yineger et al., 2008; Bekalo et al., 2009; Giday et al., 2010; Eshete, 2011; Teklay et al., 2013; Enyew et al., 2014; Mesfin et al., 2014; Kassa et al., 2016; Chekole, 2017; Teklehaymanot, 2017; Gijan and Dalle, 2019; Karakaya et al., 2019; Tewelde and Mesfin, 2019; Tamene, 2020; Tewelde, 2020; Ali et al., 2021; Assefa et al., 2021; Hankiso et al., 2024; Nuro et al., 2024) that could be attributed to their pharmacological effect and/or wider distribution.

According to the results of the current study, oral administration of medicinal plants was the primary mode of administration. Traditional healers advised their patients to take the necessary precautions when administering medicinal plants, by oral route since there is possibility that the patient will experience short- and long-term health complications. When using herbal remedies externally through the skin as opposed to internally through the mouth, there was comparatively less chance of poisoning from improper use (Giday et al., 2007). Therefore, it is imperative to prioritize the development of standardized traditional treatment guidelines pertaining to the use of medicinal plants. Oral administration was also reported by other similar studies in Ethiopia (Tolossa et al., 2013; Chekole, 2017; Fenetahun et al., 2017; Eshete and Molla, 2021) and elsewhere in the world (Guler et al., 2021; Karakose, 2022).

In the study district, leaf was the most commonly used plant part in the preparation of traditional remedies. Other ethnobotanical studies conducted in other parts of Ethiopia (Tolossa et al., 2013; Kassa et al., 2020) and elsewhere in the world (Akbulut et al., 2019; Guler et al., 2021; Karakose, 2022; Şen et al., 2022) also reported the frequent use of leaves in the preparation of remedies. The preference for leaves in remedy preparations could partly be related to their better availability. Harvesting leaves poses negative effect against survival of a plant compared to other parts (Seifu et al., 2004; Lulekal et al., 2008). The dominance of leaves could also be attributed to the production of higher concentrations of chemical compounds in the form of biologically active secondary metabolites to defend themselves from herbivores and different pathogens (Takele, 2018).

Crushing was the most commonly used method in the preparation of remedies in the study area, which is consistent with earlier reports of studies carried out elsewhere in the country (Chekole, 2017; Bekele et al., 2022). Freshly collected plant parts were predominantly used in the preparation of remedies in the area. The use of freshly harvested medicinal plant parts may be associated with the perception that such parts have a higher efficacy attributed to containing better amount of bioactive ingredients (Sofowora, 1982). Studies conducted in other parts of Ethiopia (Giday et al., 2010; Kidane et al., 2018; Kassa et al., 2020) also reported the common use of fresh plant parts in remedy preparation.

Inconsistency in dosage prescriptions of plant remedies used against same or similar ailments in the study area was frequently noted, which could be considered as one of the predicaments of traditional medicine. A study conducted elsewhere in the country also reported similar finding (Giday et al., 2009).

Among the human diseases categories reported, the high ICF value was scored for gastrointestinal problems (0.83). Similarly, of the livestock disease categories, the highest ICF value was scored for skin diseases (0.89), demonstrate the better agreement among informants in the selection of certain medicinal plants, which might be considered as indication of the potency of the plants used in treating such disorders.

In the study area, the highest ROP values (100 for each plant) were obtained for *D. torrida*, *Artemisia absinthium*, *B. aegyptiaca*, *Combretum pisoniiflorum*, *C. africana*, *O. gratissimum* and *Lagenaria* sp. for their uses against snake poison, tuberculosis, liver disorder, stomachache, tuberculosis, febrile illness and liver disorder, respectively. *Dombeya torrida* (Bekele et al., 2022), *A. absinthium* (Buffon et al., 2018), *B. aegyptiaca* (Chothani and Vaghasiya, 2011), *C. pisoniiflorum* (Danton et al., 2019) *C*. *africana*, (Getachew et al., 2022), *O. gratissimum* (Tedila and Dida, 2019) and *Lagenaria* sp. (Govind, 2011 were also reported to have same traditional medical application elsewhere in Ethiopia and other parts of the world. The anti snake poison activity of *D. torrida* may be attributed to the presence of friedelin (a triterpenoid compound), the anti-venom potency of which was revealed in a study lead by Mathias et. (2016). Extracts of the root and aerial parts of *A. absinthium* demonstrated good antibacterial effect against *Mycobacterium tuberculosis* H37Ra (Trifan et al., 2022), which may relate to the presence of bitter sesquiterpenoid lactones, flavonoids, other bitterness-imparting compounds, azulenes, phenolic acids, tannins and lignans (Szopa et al., 2020). A study conducted in Egypt on aqueous extract of the bark of *B. aegyptiaca* exhibited the high anti-hepatitis virus potential of the plant (Hafez et al., 2015). Furthermore, *in vivo* studies conducted on mice demonstrated significant hepatoprotective effect of the root bark (Traoré et al., 2020) and fruits *Balanites aegyptica* (Zein et al., 2024), which may be attributed to the presence of oil which contains mainly palmitic, stearic, oleic, and linoleic acids (Chothani and Vaghasiya, 2011). Evidence obtained from an *in vivo* study shows a significant analgesic activity of the leaf extract of *C. pisoniiflorum* (Ojewole, 2008). A related species (*Combretum micranthum*) was reported to have analgesic property due to the presence of alkaloids (Abdullahi et al., 2014) The phytochemicals kaempferol, myricetin, quercetin, and eugenol, isolated from *C. africana*, were reported to demonstrate promising antimicrobial activity. Another study demonstrated the antipyretic effect of the methanolic extracts of the leaves of *O. gratissimum* that were significant from 200 mg/kg to 300 mg/kg of body weight (Gege-Adebayo et al., 2013), the efficacy of which may be attributed to the presence of flavonoids that were previously reported to have antipyretic effect (Boakye-Gyasi et al., 2011; Achuta et al., 2011). The anti liver disorder property of *Lagenaria* sp. could be due the presence of phenolic compounds suggested to possess antioxidant activity (Thupakula and Rakesh, 2023).

In the Heban-Arsi district, it was discovered that men had better knowledge on the use of medicinal plants than women and that could be attributed to the choice of most traditional healers to transfer their knowledge on the use of medicinal plants along the male line, preferably through their first son. The traditional medical knowledge transfer through the male line is also a common occurrence elsewhere in the country (Gidey et al., 2015; Woldemariam et al., 2021).

The fact that traditional healers possess better knowledge on the use of medicinal plants shows the unevenly distribution of the knowledge among community members in the study area. Study conducted elsewhere also revealed the better knowledge on the use of medicinal plants by traditional healers compared to general informants (Woldemariam et al., 2021). Likewise, older informants mentioned higher number of medicinal plants than younger ones, which is in agreement with results of studies conducted elsewhere in the country (Giday et al., 2009; Kidane et al., 2018; Kassa et al., 2020). The better knowledge on the use of medicinal plants by older informants as compared to that of the younger ones might suggest that the latter are not interested in learning and practicing traditional medicine, the trend of which may contribute towards the deterioration of the knowledge. The study further revealed that illiterate informants reported more number of medicinal plants compared to literate informants, which is in agreement with that of Kassa et al. (2020) and that could be due to the fact that literate people are more exposed to Western modernization and as a result consider traditional medical as backward and harmful.

The majority of medicinal plants were reported to be harvested from the wild, which is consistent with results of studies conducted elsewhere in Ethiopian (Awas and Demissew, 2009; Kidane et al., 2018; Gijan and Dalle, 2019) which could be showing the poor culture of people in cultivating medicinal plant. This is very worrying in the face of the ongoing habitat destruction in the study area mainly due to clearing of forests for more agricultural lands.

## 5 Conclusion

Medicinal plants in Heban-Arsi district still play a vital role in fulfilling the primary healthcare needs of community members as revealed by the high diversity of the reported medicinal plant species. Among the various ailments categories, human gastrointestinal disorders and skin disorders of animals scored the highest ICF values. Of all reported medicinal plants, *B. aegyptica* and *D. torrida* exhibited the highest fidelity level values in treating liver disease and snakebite poisoning, respectively. The factors that posed a higher threat to survival of medicinal plants and the associated knowledge in the area included population growth, poverty, forests clearing for more agricultural lands, the secrecy and oral transmission of knowledge. It is thus imperative to develop conservation strategy for the traditional medical practice to continue in the study area. It is recommended that attention for further bioactivity and phytochemical investigations should be given to medicinal plants reported to be used against diseases categories of high ICF values and those that scored the highest FL and preference ranking values.

## Data Availability

The original contributions presented in the study are included in the article/Supplementary Material, further inquiries can be directed to the corresponding author.

## Abbreviations

**AAU**, Addis Ababa University; **ALIPB**, Aklilu Lemma Institute of Pathobiology; **ALIPB-IRERC**, Aklilu Lemma Institute of Pathobiology Institutional Research Ethics Review Committee; **CSA**, Central Statistics Agency; **FL**, Fidelity level; **ICF**, Informant consensus factor; **ROP**, Rank order priority; **RPL**, relative popularity level; **WHO**, World Health Organization
