# Supplementary material for: Medicinal plants diversity among the oromo community in heban-arsi district of Ethiopia used to manage human and livestock ailments
Source: Front Pharmacol. 2024 Sep 19;15:1455126. doi: 10.3389/fphar.2024.1455126 (PMC11450779; doi:10.3389/fphar.2024.1455126)
Supplement: Supplementary file 1 [file Table1.docx]

**Supplementary Table 1. Medicinal plants used to treating human and livestock diseases in Heban-Arsi district, Ethiopia**

**L: leaves, Ba: bark, R: root, Wh; whole part; St: stem; Fr: fruit; Se: seed; Sa: Sap; La: latex; Epi: epiphyte; Sh: Shoot; Ap: apex**

| Scientific Name | Family | Local Name | PU | disease | Treated | Mode of preparation | Administration route | No of use reports | Voucher number |
| --- | --- | --- | --- | --- | --- | --- | --- | --- | --- |
| *Acacia* sp. | Fabaceae | dhadacha | R | Body swelling | Hu | Grind and apply as poultice | Topical | 5 | GN-103 |
| *Acacia* sp. | Fabaceae | garbii | Ba | wound | Ls | Crush, dry and grind to make powder | Topical | 6 | GN-171 |
| *Achyranthes aspera* L. | Amaranthaceae | Dargu | L | Skin diseases | Hu | Heat, grind, and mix powder with butter | External | 9 | GN-21 |
|  |  |  | L | Pneumonia | Hu/Ls | Drink decoction | Oral | 14 |  |
|  |  |  | L | Body swelling | Hu | Squeeze & apply paste on the swollen part | Topical | 7 |  |
|  |  |  | L | Stomach ache | Hu | Chewed fresh and swallow when pain is feeling | oral | 13 |  |
|  |  |  | L | Throat pain | Hu | Squeeze and drink extract | Oral | 12 | GN-120 |
|  |  |  | R/L | Aspiration | Hu/Ls | Crush & smash with teeth | Oral for Hu & nostrils for LS | 28 |  |
| *Acokanthera schimperi* (A.DC) Schweinf | Apocynaceae | Qaraaru | R | malaria | Hu | Crush fresh and mix with water | Oral | 61 | GN-178 |
|  |  |  | L | Spider poison | Hu | Crush fresh, squeezed and wash with liquid produced | Topical | 2 |  |
|  |  |  | L | Dermatophilosis | Hu/Ls | Apply ground leaves mixed with butter | Topical | 1 |  |
| *Adiantum* sp. | Adiantaceae | Laaftu | L | Stomach ache | Hu | Apply fresh ground leaves | Oral | 6 | GN-72 |
| Afrocarpus falcatus (Thunb.) C.N.Page | Podocarpaceae | Birbirsa | L | Cancer | Hu | Decoction | Oral | 21 | GN-8 |
| *Ajuga integrifolia* Buch.-Ham. ex D.Do | Lamiaceae | Harmaguusa | L | Stomach ache | Hu | Crush & mix with water | Oral | 8 | GN-182 |
| Alantsilodendron pilosumV illiers | Fabaceae | Geeto | Ba | Dermatophilosis | Ls | Crushed and boiled in water | Body wash | 12 | GN-129 |
| *Albizia gummifera* (J.F.Gmel.) C.A.Sm | Fabaceae | Qarcacee | Ba | Aspiration | Hu | Internal part of bark is chewed & swallowed | Oral | 46 | GN=88 |
|  |  |  | Ba | Anthrax | Ls | Fresh bark is crushed & mixed with water | oral | 4 |  |
| *Albizia schimperiana* Oliv. | Fabaceae | Sisaa | L | Pneumonia | Ls | Fresh leaves are crushed & juice produced | Oral drench | 33 | GN-43 |
|  |  |  | L | Stomach ache | Hu | Fresh leaves are crushed & juice is extracted | Oral | 6 |  |
|  |  |  | L | Dry skin | Hu | Rub crushed leaves | Topical | 3 |  |
|  |  |  | Fr | Syphilis | Hu | Dry, grind and mix with water | Oral | 3 |  |
| *Allophylus abyssinicus* (Hochst.) Radlk | Sapindaceae | Hirqamu | Ba | wound | Hu | Scrape bark and apply extract | External | 9 | GN-61 |
|  |  |  | L | Hemorrhoids | Hu | Fresh leaves are crushed & paste applied | Topical | 19 |  |
|  |  |  |  | diarrhea | Hu | Decoction | Oral | 21 |  |
| *Aloe* sp. | Aloaceae | hargisa | L | Diarrhea | Hu | Crush and sap obtained is mixed with water | Oral | 31 | GN-127 |
|  |  |  | L | Hair loss | Hu | Crush & apply | Topical | 50 |  |
|  |  |  | L | Sudden sickness | Ls | Crush and mix with water | Oral drench | 6 |  |
| Amaranthus tortuosus Hornem. | Amaranthaceae | Raafu | Wh | Constipation | Hu | Fresh part is crushed and boiled | Oral | 11 | GN-168 |
| *Ampelocissus* sp*.* | Vitaccae | laalu | L | Liver | Hu | Crushed and boiled | Oral | 23 | GN-137 |
|  |  |  | R | Hemorrhoids | Hu | Root is pounded and mixed with *Allium sativa* L | Apply as poultice | 17 |  |
| Artemisia absinthium L. | Asteraceae | Ariitii | L | TB | Hu | Decoction | Oral | 47 | GN-183 |
| Astropanax abyssinicus (Hochst. ex A.Rich.) Seem. | Araliaceae | Gatame | Ba | Tonsillitis | Hu | Crushed, chewed and ingested | Oral | 18 | GN-151 |
|  |  |  | Ba | Liver disorders | Hu | Crushed & boiled | Oral | 9 |  |
|  |  |  | Ba | Anthrax | Ls | Crushed & boiled | Oral drench | 11 |  |
| Astropanax volkensii (Harms) Lowry, G.M.Plunkett, Gostel & Frodin | Araliaceae | Ansha | Ba | Tonsillitis | Hu | Inner bark chewed & swallowed | Oral | 6 | GN-161 |
| *Balanites aegyptiaca* (L.) Del. | Balanitaceae | Bedana | R | Liver disorder | Hu | Fresh roots crushed, squeezed & boiled | Oral | 46 | GN-105 |
| *Bersama abyssinica* Fresen. | Melianthaceae | Koraqa | Ba | Internal parasite | Hu | Crushed and mixed with water | Oral | 17 | GN-1 |
|  |  |  | L |  | Ls | Crushed and mixed with water | Oral | 15 |  |
|  |  |  | L | Pneumonia | Hu | Apply crushed leaf extract | Oral | 9 |  |
|  |  |  |  | Diarrhea | Hu | Crushed and boiled | Topical | 13 |  |
| Biancaea decapetala (Roth) O.Deg. | Fabaceae | Gorxa | St | Evil eye | Ls | Fresh stem is rolled around neck | External | 7 | GN-35 |
| *Brassica* sp. | Brassicaceae | Gomana | Wh | Stomach ache | Hu | Fresh part crushed and boiled | Oral | 6 | GN-131 |
| *Brucea antidysenterica* JF. Mill. | Simaroubaceae | Ciironta | L | Dermathophilosis | Hu | Leaf dried and smoked | Nostrils | 22 | GN-26 |
|  |  |  | L | Evil eye | Ls | Fresh part crushed and mixed with water | Oral | 4 |  |
|  |  |  | L | Diarrhea | Hu/Ls | Crushed and squeezed | Oral | 31 |  |
|  |  |  | Ba | External parasite | Ls | Scrape bark and apply | External | 11 |  |
| *Buddleja polystachya* Fresen*.* | Loganiaceae | Bulchaana | Ba | wound | Hu/Ls | Dried and powdered mixed with butter | Topical | 25 | GN-158 |
|  |  |  | L | Dermatophilosis | Ls | Crushed & mixed with water | Topical | 10 |  |
|  |  |  | L | Tonsillitis | Hu | Crushed, squeezed & extract drunk | Oral | 8 |  |
| *Cadaba farinosa* Forssk | Capparidaceae | Qalqalcha | L | External parasite | Hu/Ls | Fresh part crushed & pounded | Dermal | 9 | GN-112 |
|  |  |  | St | Ringworm | Hu | Fresh stem crushed and boiled in water | Topical | 3 |  |
| *Calpurnia aurea* (Aiton) Benth. | Fabaceae | Ceekata | R | Liver disease | Hu | Crushed, dried & powdered & mixed with water | Oral | 39 | GN-143 |
|  |  |  | L | Wound | Hu/Ls | Crushed, dried & powdered | Topical | 27 |  |
|  |  |  | Sh | Diarrhea | Hu | Fresh shoot chewed with salt | Oral | 21 |  |
|  |  |  | L | Skin diseases scabies/lice) | Ls | Dried, pounded mixed with honey | Dermal | 51 |  |
| *Capparis tomentosa* Lam. | Capparidaceae | Hobe mada | Ba | Babesiosis | Ls | Crushed and dried and powder mixed with water | Oral drench | 9 | GN-81 |
|  |  |  | Ba | Depression | Hu | Fresh bark smashed & smoked | Nostrils | 13 |  |
|  |  |  | Rb | Eczema | Hu | Fresh root bark crushed squeezed & juice extracted | Topical | 4 |  |
|  |  | Hunxuuxi | L | Male erectile problem | Hu | Fresh leaves crushed and squeezed & juice mixed with water | Oral | 37 | GN-102 |
|  |  |  | R | Tumor | Hu | Crushed and pounded | Poultice | 24 |  |
|  |  |  | R | Tumor | Ls | Crushed and pounded | Topical | 10 |  |
|  |  |  | Fr | Diabetes | Hu | Ripe fruit eaten | Oral | 33 |  |
| *Carissa spinarum* L. | Apocynaceae | Agamsa | R | Cancer | Hu | Crushed, dried and powdered with root of *Euclea rochetinia* & boiled | Orally | 26 | GN-23 |
|  |  |  | R | Toothache | Hu | Fresh root crushed | Kept between teeth | 5 |  |
|  |  |  | R | Evil eye | Hu | Freeh root crushed and mixed with water | Oral | 19 |  |
| *Cassipourea malosana* (Baker) Alston | Rhizophoraceae | Xiiloo | Ba | wound | Ls | Bark dried, powdered & mixed with honey | Dermal | 8 | GN-156 |
| *Celtis africana* Burm. f | Ulmaceae | Amalaqa | Ba | Tuberculosis | Hu | Crushed, dried, powdered and mixed with water & boiled | Oral | 30 | GN-89 |
|  |  |  | Ba | Tuberculosis | Ls | crushed | Oral | 8 |  |
| Coleus abyssinicus (Fresen.) A.J.Paton | Lamiaceae | Ajaawa | L | Eye pain | Hu/Ls | Crushed and squeezed | Topical | 1 | GN=179 |
| Combretum pisoniiflorum (Klotzsch) Engl. | Combretaceae | Rukeessa | L | Stomach ache | Hu | Fresh leaves crushed & mixed with water | Oral | 40 | GN-167 |
| *Croton dichogamus* Pax | Euphorbiaceae | Ulee foonii | L | Malaria | Hu | Fresh leaves crushed, squeezed & mixed with water | Oral | 23 | GN-114 |
|  |  |  | L | Anthrax | Ls | Crushed and mixed with water | Oral | 19 |  |
| *Croton macrostachyus* Hochst. | Euphorbiaceae | Mokoniisa | Ba | wound | Hu/Ls | Crushed, dried, powdered and mixed with butter | Topical | 15 | GN-25 |
|  |  |  | Ba | Dermatophilosis | Hu/Ls | Crushed, dried, powdered and mixed with butter | Topical | 21 |  |
|  |  |  | L | Hemorrhoids | Hu | Crushed, dried, powdered and mixed with butter | Topical | 11 |  |
|  |  |  | L | Liver disorder | Hu | Crushed, dried, powdered and boiled | Oral | 23 |  |
| *Datura stramonium* L. | Solanaceae | Banji | R | Rabies | Hu/Ls | Crushed squeezed & mixed with water | Oral | 35 | GN-165 |
|  |  |  | L | Lumpy skin disease | Ls | Crushed & squeezed | Dermal | 11 |  |
| *Discopodium penninervum* (Hochst.) | Solanaceae | Maraaroo | L | bloat | Ls | Crushed, squeezed & mixed with water | Oral | 15 | GN-29 |
|  |  |  | L | Liver disorders | Hu | decoction | Oral | 6 |  |
| *Dodonaea viscosa* subsp. *angustifolia* (L.f.) J.G.West | Sapindaceae | Itacha | L | Dermatophilosis | Hu/Ls | Dried and pounded | Topical | 43 | GN-136 |
|  |  |  | Ba | wound | Hu | Dried and pounded | Topical | 9 |  |
|  |  |  | L | Diarrhea | Hu | Crushed & squeezed | Oral | 20 |  |
| *Dombeya torrida* (J.F. Gmel.) Bamps | Euphorbiaceae | Daanisa | L | Snake poison | Hu | Squeezed and mixed with water | Oral | 57 | GN-164 |
| *Echinops angustilobus* S.moore | Asteraceae | Anshokala | L | Bad sprit | Hu | Crushed and squeezed | Spray | 6 | GN-153 |
|  |  |  |  | Bloat | Ls | Crushed & mixed with water | Oral | 1 |  |
| *Ekebergia capensis* Sparrm. | Meliaceae | Onoonu | L | Child emaciation | Hu | Crushed & boiled in water | Oral | 16 | GN-55 |
| *Englerina woodfordioides* (Schweinf) M Gilbert | Loranthaceae | Mukuree | Epi | Liver disease | Hu | Crushed, squeezed and mixed with water & boiled | Oral | 21 | GN-36 |
| *Ensete ventricosum* (Welw.) Cheesman | Musaceae | Ensat | St | Stomach ache | Hu | Crushed & fermented | Oral | 6 | GN-94 |
| *Erucastrum* sp. | brassicaceae | Sharalee | L | Stomach ache | Hu | Crushed & boiled | Oral | 3 | GN-101 |
|  |  |  | L | Febrile sickness | Hu | Fresh leaf chewed & swallowed | Oral | 4 |  |
| *Erythrina brucei* Schwein. | Fabaceae | Woleena | L | Eye pain | Ls | Crushed, squeezed & extracted | Spray | 25 | GN-5 |
| *Eucalyptus camaldulensis* Dehnh. | Myrtaceae | Bargamo diima | L | Cough | Hu | Crushed and macerated | Nostrils | 2 | GN-159 |
| *Euclea racemosa* L. | Ebenaceae | Mi’eesa | R | Skin disorder | Hu | Crushed, dried, powdered and mixed with butter | Topical | 30 | GN-87 |
|  |  |  | L | Tooth ache | Hu | Crushed | Kept between tooth | 13 |  |
| Euphorbia schimperiana var. schimperiana | Euphorbiaceae | Guurii | Sap | hemorrhoids | Hu | Crushed & Squeezed | Topical | 12 | GN-6 |
|  |  |  | Lat | Syphilis | Hu | Crushed & Squeezed | Topical | 34 |  |
| *Euphorbia tirucalli* L. | Euphorbiaceae | Ananoo | Sa | Snake bite | Hu/Ls | Crushed and juice extracted | Oral | 9 | GN-100 |
| *Ficus glumosa* Delile | Moraceae | Dambii | L | Anthrax | Ls | Crushed squeezed & mixed with water | Oral | 7 | GN-157 |
|  |  |  | L | Constipation | Hu | Fresh leaf crushed and squeezed & macerated | Oral | 3 |  |
| *Ficus ruspolli* Warb*.* | Moraceae | dadhi | L | Stomach ache | Hu | decoction | Oral | 1 | GN-184 |
| *Ficus* sp. | Moraceae | Qilxuu | Ba | Diarrhea | Hu | Crushed & extracted to make juice | Oral | 10 | GN-104 |
| *Ficus sur* Forssk. | Moraceae | Oddaa | Fr | Stomach ache | Hu | Dried, powdered and mixed with water | Oral | 11 | GN-66 |
| *Galiniera saxifraga* Hochst.ex.Bridson | Rubiaceae | Koralla | L | Malaria | Hu | Squeezed and mixed with water | Oral | 2 | GN-15 |
|  |  |  | Ba | TB | Hu | Crushed & boiled in water | Oral | 28 |  |
|  |  |  | L | Anthrax | Ls | Crushed & mixed with water | Nostrils | 7 |  |
| *Galium* sp. | Rubiaceae | Maxxane | Wh | Evil eye | Ls | Crushed and mixed with water | Spray | 2 | GN-98 |
| *Gomphocarpus integer* (HE. Br.) Bullock | Asclepiadaceae | Haari hiyo | R | Syphilis | Hu | Crushed & latex produced | Topical | 3 | GN-123 |
| *Grewia ferruginea* Hochst. ex A. Rich. | Tiliaceae | Dhoqona | L | Diarrhea | Ls | Fresh leaf crushed & mixed with water | Oral | 3 | GN-154 |
| *Grewia mollis* A. Juss. | Tiliaceae | Harooressa | R | Head ache | Hu | Dried root boiled | Oral | 1 | GN-118 |
| *Grewia villosa* Willd. | Tiliaceae | mandhera | L | Head ache | Hu | Crushed & smelled | Nostrils | 4 | GN-115 |
|  |  | Ogobdi | L | Diarrhea | Hu | Crush and boiled | Oral | 1 | GN-119 |
| Gymnanthemum amygdalinum (Del.) Sch.Bip. ex Walp. | Asteraceae | Ebicha | L | malaria | Hu | Fresh leaves crushed & mixed with water | Oral | 14 | GN-12 |
|  |  |  | L | Stomach ache | Ls | Smashed & mixed with water | Oral | 33 |  |
| Gymnanthemum auriculiferum (Hiern) Isawumi | Asteraceae | Reeji | Epi | Skin cancer | Hu | Crashed and boiled | Dermal | 3 | GN-13 |
|  |  |  | Ap | Bloat | Ls | Crushed & mixed with water | Oral | 37 |  |
|  |  |  | R | Sexually transmitted diseases | Hu | Decoction | Oral | 5 |  |
| Gymnosporia addat Loes. | Celasteraceae | Kombolcha | St | Evil eye | Ls | Fresh stem placed on the neck | Topical | 3 | GN-53 |
| *Hagenia abyssinica* (Brace) JF. Gmel. | Rosaceae | Heexoo | Fr | Internal parasite | Hu | Dried, grounded & mixed with water | Oral | 40 | GN-91 |
| *Halleria lucida* L. | Scrophulariaceae | Muka dadhii | L | Epilepsy | Hu | Fresh leaf is crushed and it’s juice is mixed with water | Oral | 4 | GN-54 |
| *Hypericum revolutum* Vahl | Hypericaceae | Garamba | L | Liver disease | Hu | Fresh leaf crushed, pounded and mixed in water | Oral | 13 | GN-45 |
|  |  |  | L | Pasterollosis | Ls | Fresh leaf crushed, pounded and mixed in water | Oral | 9 |  |
| *Ilex mitis* (L.) Radlk | Aquifoliaceae | Amshiqa | Ba | Hemorrhoids | Hu | Fresh bark decoction | Oral | 11 | GN-49 |
| *Juniperus procera* Hochst. ex Endl. | Cuperssaceae | Hindheesa umam maa | Fr, L | Abnormal menstrual period | Hu | Decoction | Oral | 15 | GN-14 |
|  |  |  | L | Emaciated child | Hu | Fresh leaf crushed, mixed with water and boiled | Oral | 3 |  |
|  |  |  | Fr | Skin diseases | Hu | Roasted over hot plate, grounded & mixed with butter | Topical | 3 |  |
| *Kalanchoe laciniata* (L.) DC. | Crassulaceae | Hancuura gamoji | R | Tonsillitis | Hu | Fresh root crushed & ingested | Oral | 13 | GN-166 |
| *Lagenaria* sp. | Cucurbitaceae | Buqe | Fr | Liver disorder | Hu | Crushed and liquid collected | Oral | 32 | GN-67 |
| *Laggera crispata* (Vahl) Hepper & Wood | Asteraceae | Ajjeesa | Wh | Bloat | Ls | Crushed, squeezed & mixed with water | Oral | 1 | GN-144 |
| *Lantana camara* L. | Verbenaceae | Qorso sarti | L | wound | Hu | Crushed, squeezed and mixed with water | Dermal | 1 | GN-82 |
| *Maesa lanceolata* Forssk. | Myrsinaceae | Abbayi | Ba | Dermatophilosis | Hu/Ls | Fresh bark crushed & mixed with water | Dermal | 16 | GN-142 |
|  |  |  | La | Skin disease | Hu | Crushed, dried & powdered | Dermal | 19 |  |
|  |  |  | L | Diabetes | Hu | Decoction | Oral | 10 |  |
|  |  | Arabdotesa | Ba | Emaciated child | Hu | Bark is crushed & boiled | Body wash and oral | 3 | GN-155 |
| *Malus sylvestris* Mill. | Rosaceae | Appili | Fr | Hemorrhoids | Hu | Smashed | Oral | 12 | GN-109 |
| *Mangifera indica* L. | Anacardiaceae | Mango | Fr | Gastritis | Hu | Squeezed & juice extracted | Oral | 10 | GN-110 |
| *Melia azedarach* L. | Meliaceae | Neemii | Sh | Febrile sickness | Hu | Fresh leaves crushed, squeezed & mixed with water | Oral | 10 | GN-138 |
|  |  |  | L | Liver disorders | Hu | Fresh leaf crushed, squeezed & mixed with water | Oral | 22 |  |
|  |  |  | Fr | Internal parasites |  | Fresh fruits crushed & swallowed | Oral | 20 |  |
| *Millettia ferruginea* (Hochst.) Hochst. ex Baker | Fabaceae | Dhadhatu | St | Rabies | Ls | Stem heated and put over bitten part | dermal | 7 | GN-90 |
|  |  |  | L | Leech | Ls | Crushed and immersed in water | Oral drench | 16 |  |
| *Mimusops kummel* Bruce ex A.DC. | Sapotaceae | Olaati | Fr | Stomach ache | Hu | Ripe fruit crushed | Oral | 7 | GN-133 |
|  |  |  | Fr | Cold disease | Hu | Ripe fruits crushed | Oral | 3 |  |
| *Momordica foetida* Schumach. | Cucurbitaceae | Hoola waaqa | L | Bad sprit | Hu | Leaf pounded, burned & smoked | Nostrils | 1 | GN-79 |
| *Moringa stenopetala* (Bak. f) Cufod. | moringaceae | Moringa | L | Blood pressure & diabetes | Hu | Dried, grounded & mixed with water | Oral | 31 | GN-126 |
| *Myrica salcifolia* A.Rich. | Myricaceae | Qamoo | Ba | Prostate problem | Hu | Decoction | Oral | 5 | GN-149 |
| *Myrsine melanophloeos* (L.) R. Br. | Myrsinaceae | Tuula | L | Child emaciation | Hu | Decoction of fresh leaves | Oral & body wash | 7 | GN-41 |
| *Nicotiana tabacum* L. | Solanaceae | Baala tambo | L | Bloat | Ls | Crushed, squeezed & juice produced | Nostrils | 11 | GN-175 |
| *Nuxia congesta* R.Br. ex Fresen. | Loganiaceae | Biixana | Ba | Tonsillitis | Hu | Fresh internal part of bark chewed and swallowed | Oral | 15 | GN-47 |
| *Ocimum gratissimum* L. | Lamiaceae | Cabbicha | L | Febrile sickness | Hu | Crushed & boiled | Oral | 36 | GN-80 |
| *Ocimum lamiifolium* Hochst. ex Benth. | Lamiaceae | Damakase | L | Febrile sickness | Hu | Crushed & squeezed | Nostrils | 25 | GN-77 |
| *Oenanthe palustris* (Chiov.) Norman | Apiaceae | Goonde | L | cancer | Hu | Fresh leaves crushed & squeezed | Infused into the wound | 12 | GN-42 |
| *Olea europaea* subsp. *cuspidata* (Wall. ex G.Don) Cif | Oleaceae | Ejersa | W | Skin cancer | Hu | Cut, placed into pot & put on fire for oil production | Topical | 6 | GN-46 |
|  |  |  | wh | Rheumatism | Hu | Cut, placed into pot & put on fire for oil production | Massage | 30 |  |
|  |  |  | Sh | Tumor | Ls | Dried, powdered | Topical | 3 |  |
| *Olea welwitschii* (Knobl.) Gilg & Schellenb. | Oleaceae | Dhama’e | L | Skin disease | Hu | Fresh leaf crushed & smelled | Topical | 7 | GN-154 |
|  |  |  | Ba | Arthritis | Hu | Fresh leaf crushed, squeezed & mixed with water | Topical | 3 |  |
| *Olinia rochetiana* A. Juss. | Oliniaceae | Gunaa | L | Stomach ache | Hu | Fresh leaves crushed, squeezed & mixed with water | Oral | 47 | GN-17 |
|  |  |  | L | Cancer | Hu | Fresh leaf chewed, ingested & the residue pasted on the wound | Oral & topical | 16 |  |
|  |  |  | St | Cold disease | Hu | Cut & put on fire after placing it in pot | Fumigation | 31 |  |
|  |  |  | L | Respiratory infections | Hu | Decoction | Oral | 5 |  |
| *Physalis peruviana* L. | Solanaceae | Mujulo | Wh | Constipation | Hu | Crushed and boiled | Oral | 9 | GN-111 |
| *Phytolacca dodecandra* L 'Herit. | Phytolaccaceae | Hamdoode | L | Rabies | Hu/Ls | Crushed & mixed with water | Oral | 49 | GN-93 |
|  |  |  |  | Itching | Hu | Crushed & mixed with water | Topical | 2 |  |
| *Polyscias fulva* (Hiem) Harms | Araliaceae | Guduba | L | Bloat | Ls | Crushed and put in water to be macerated | Oral | 11 | GN-96 |
| *Premna schimperi* Engl. | Lamiaceae | Baala xoxoqe | L | Itching | Hu | Decoction | Dermal | 1 | GN-135 |
| *Prunus africana* (Hook.f) Kalkman | Rosaceae | Sukkee | Ba | wound | Hu/Ls | Fresh internal part of bark crushed & squeezed | Dermal | 40 | GN-27 |
|  |  |  | Sh | Diarrhea | Hu | Decoction | Oral | 23 |  |
| *Psydrax schimperianus* (A.Rich.) Bridson | Rubiaceae | Gaaloo | Fr | Stomach ache | Hu | Ripe fruit eaten | Oral | 2 | GN-20 |
| *Rhamnus prinoides* L 'Herit. | Rhamnaceae | Geesho | Wh | Diarrhea | Ls | Crushed and macerated | Oral drench | 5 | GN-170 |
| *Rhoicissus tridentata* (L. f) Wild & Drumm. | Vitaceae | Laalo | L | Jaundice | Hu | Decoction | Oral | 15 | GN-137 |
|  |  |  | L | Hemorrhoids | Hu | Fresh leaf crushed | Paste | 7 | GN-137 |
|  |  |  | L | Skin disease | Hu/Ls | Crushed & Squeezed | Dermal | 3 |  |
| Rotheca myricoides (Hochst.) Steane & Mabb. | Lamiaceae | Maraachisa | L | Abdominal pain | Hu | Crushed & boiled | Oral | 23 | GN-4 |
| *Ricinus communis* L. | Euphorbiaceae | Qoboo | Se | Calf diarrhea | Ls | Dried, powdered and mixed with water | Oral | 9 | GN-92 |
| *Rubus apetalus* poir. | Rosaceae | Gorra | Fr | Jaundice | Hu | Fresh ripe fruits squeezed | Oral | 2 | GN-40 |
| *Rumex nepalensis* Spreng. | Polygonaceae | Shaabe | R | Hemorrhoids | Hu | Dried, ground & mixed with water | Oral | 12 | GN-9 |
|  |  |  | R | Tumor | Hu | Dried, ground & mixed with water | Oral | 6 |  |
|  |  |  |  | Pressure | Hu | Decoction | Oral | 14 |  |
|  |  |  | L | Stomach ache | Hu | Decoction | Oral | 22 |  |
| *Ruta chalepensis* L. | Rutaceae | Caarota | Wh | Urinary infections | Hu | Maceration | Oral | 4 | GN-97 |
|  |  |  |  |  |  |  |  |  |  |
| Salvia rosmarinus Spenn. | Lamiaceae | sigamatibasha | Wh | Blood pressure | Hu | Decoction | Oral | 10 | GN-185 |
| Scepocarpus hypselodendron (Hochst. ex A.Rich.) T.Wells & A.K.Monro | Urticaceae | Haliila | St | Diarrhea | Ls | Cut into pieces & immersed in water | Oral drench | 4 | GN-31 |
| *Schrebera alata* (Hochst.) Welw. | Oleaceae | Siigeda | L | Anthrax | Ls | Crushed and mixed with water | Oral | 3 | GN-150 |
| Searsia natalensis (Bernh. ex C.Krauss) F.A.Barkley | Anacardiaceae | Xaxeessa | L | Stomach ache | Ls | Pounded and juice produced | Oral | 2 | GN-113 |
| *Senecio myriocephalus* Sch. Bip. ex A. Rich. | Asteraceae | Agadena | L | wound | Hu | Crushed & paste put on the infected part | Dermal | 2 | GN-163 |
| *Sida rhombifolia* L. | Malvaceae | Kotejabeesa | R | Cough | Hu | Dried, powdered and mixed with water | Oral | 3 | GN-152 |
| *Solanecio gigas* (Vatke) C. Jeffrey | Asteraceae | Taruura | R | Stomach ache | Hu | Crashed & mixed with water | Oral | 5 | GN-!72 |
| *Solanum anguivi* Lam. | Solanaceae | Hiddi oromo | L | Hemophilia | Hu | Fresh leaves crushed | Inhale | 13 | GN-10 |
| *Stephania abyssinica* (Dillon & A.Rich.)Walp | Menispermaceae | Kalaala | St | Jaundice | Hu | Fresh stem crushed & mixed with water | Oral | 22 | GN-11 |
|  |  |  | Wh | Difficulty in urination | Ls | Rolled around the neck | External | 20 |  |
| *Syzygium guineense* (Willd.) DC. | Myrtaceae | Badeesa | L | Diarrhea | Hu | Fresh leaves crushed, squeezed & mixed with water | Oral | 52 | GN-62 |
|  |  |  | L | Cough |  | Leaves boiled | Oral | 4 |  |
| *Tragia* sp. | Euphorbiaceae | Laleesa | L | bloat | Ls | Crushed, squeezed & mixed with water | Oral | 16 | GN-116 |
| *Vachellia etbaica* (Schweinf.) Kyal. & Boatwr. | Fabaceae | dodota | Ba | Skin diseases | Hu/Ls | Crush fresh and boil | Topical | 7 | GN-119 |
| Vachellia tortilis (Forssk.) Galasso & Banfi | Fabaceae | Harbuu | L/Ba | wound | Ls | Crush, dry and grind to make powder | Topical | 14 | GN-173 |
|  |  |  | L/Ba | wound | Ls | Crush, dry and grind to make powder | Topical | 14 |  |
| Vepris nobilis (Delile) Mziray | Rutaceae | Hadhesa | L | Tonsillitis | Hu | Smashed & chewed | Oral | 2 | GN-30 |
| *Vernonia noveboracensis* (L) Michx. | Viscaceae | Hincici | W | Liver disease | Hu | Smashed, dried and boiled | Oral | 1 |  |
| *Viscum* sp. | Viscaceae | Bilbileti |  | Liver disorder | Hu | Smashed, dried and boiled | Oral | 2 | GN-174 |
| *Withania somnifera* (L.) Dunal | Solanaceae | Baala | Fr | Evil eye | Hu | Crushed & squeezed | Spray | 22 | GN-84 |
|  |  | Ajo | L | Stomach ache | Hu | Crushed & mixed with water | Oral | 34 |  |
| *Ximenia americana* L. | Olacaceae | Hudha | L | wound | Hu | Crushed, dried & mixed with butter | Topical | 7 | GN-124 |
|  |  |  | Ba | Diarrhea | Hu | Crushed, dried, pounded & boiled | Oral | 26 |  |
| Zanthoxylum asiaticum (L.) Appelhans, Groppo & J.Wen | Rutaceae | Gawo | Ba | Stabbing pain | Hu | Crushed, mixed with water & boiled | Oral | 11 | GN-28 |
| *Ziziphus* *spina-christi* (L.) Desf. | Rhamnaceae | Qurqura | L | Stomach ache & diarrhea | Hu | Ripe fruit squeezed & juice collected | Oral | 21 | GN-99 |
